# Abdominal obesity among Chinese children and adolescents: a systematic review and meta-analysis of prevalence and study-level heterogeneity

**DOI:** 10.3389/fnut.2026.1839333

**Published:** 2026-07-29

**Authors:** Bo Chen, Xufeng Xu, Zisheng Li, Jiaqing Dou

**Affiliations:** 1 Department of Nuclear Medicine, The Fourth Affiliated Hospital of Anhui Medical University, Hefei, Anhui, China; 2 Department of Traditional Chinese Medicine (TCM) Surgery, The Fourth Affiliated Hospital of Anhui Medical University, Hefei, Anhui, China; 3 Department of Endocrinology, The Fourth Affiliated Hospital of Anhui Medical University, Hefei, Anhui, China

**Keywords:** abdominal obesity, children and adolescents, China, Meta-analysis, nutritional epidemiology, prevalence, systematic review

## Abstract

**Background:**

Abdominal obesity in youth is a nutrition-related public health concern and may capture cardiometabolic risk beyond BMI. We aimed to estimate the pooled prevalence of abdominal obesity among Chinese children and adolescents and explore study-level heterogeneity.

**Methods:**

PubMed, Web of Science, Embase, CNKI, WanFang, and VIP were searched from inception to December 31, 2025. Eligible studies reported abdominal obesity prevalence among children and adolescents in China. Random-effects meta-analysis of proportions was performed. Subgroup analyses and exploratory meta-regression assessed variation by survey year, sex, urban–rural setting, diagnostic criterion, and study quality.

**Results:**

Twenty-eight studies contributing 30 prevalence estimates were included. The pooled prevalence was 20.0% (95% CI: 19.0–22.0%; 95% prediction interval: 11.9–31.3%), with very high heterogeneity (I^2^ = 99.54%, *p* < 0.001). Pooled estimates were higher in urban than rural populations (22.8% vs. 16.7%) and in male than female strata (23.1% vs. 16.4%), while estimates were similar between waist-to-height ratio and waist circumference criteria. Survey year was not significantly associated with prevalence in linear or spline-based meta-regression, and multivariable meta-regression did not identify significant study-level predictors.

**Conclusion:**

Standardized waist-based surveillance, alongside BMI, is needed to improve comparability across studies and to support school- and community-based prevention strategies targeting central adiposity in Chinese youth.

**Systematic Review Registration:**

https://www.crd.york.ac.uk/PROSPERO/view/CRD420251053503, identifier PROSPERO (CRD420251053503).

## 1 Introduction

Childhood and adolescent obesity has become a major global public health challenge. Recent evidence indicates that pediatric obesity remains highly prevalent worldwide and continues to rise in many settings, with important consequences for metabolic, cardiovascular, and psychosocial health (1, 2). Obesity that develops early in life is also associated with an elevated risk of insulin resistance, dyslipidemia, hypertension, and long-term cardiometabolic disorders (2).

Compared with general obesity defined by body mass index (BMI), abdominal obesity reflects central fat accumulation more directly and may better capture obesity-related cardiometabolic risk (3). In children and adolescents, abdominal obesity is commonly assessed using waist circumference (WC) and waist-to-height ratio (WHtR), both of which are simple and feasible anthropometric indicators for epidemiological surveys and clinical practice (3). Recent evidence suggests that WHtR, WC, and BMI all have value in identifying clustered cardiometabolic risk in young populations, while WHtR may be particularly practical for population screening because of its simplicity (3). In this sense, abdominal obesity is not only an anthropometric phenotype but also a nutrition-relevant marker of adverse metabolic health in young populations, especially in settings undergoing rapid dietary and lifestyle change.

China provides an important context for studying abdominal obesity in youth. Over recent decades, rapid urbanization, economic development, and nutrition transition have reshaped diet quality, food environments, physical activity, sleep, and sedentary behavior, contributing to a growing obesity burden (4). National evidence has shown that overweight and obesity have become major public health concerns in China, driven by shifts toward energy-dense diets, increasing consumption of sugar-sweetened beverages and ultra-processed foods, reduced habitual physical activity, and broader changes in the obesogenic environment (4). In this context, abdominal obesity among Chinese children and adolescents deserves particular attention because central adiposity may provide additional information beyond BMI alone for identifying early cardiometabolic risk and guiding nutrition surveillance and prevention (4, 5).

Although an increasing number of studies have reported the prevalence of abdominal obesity among Chinese children and adolescents, the available evidence remains fragmented. Individual studies differ substantially in geographic coverage, sampling design, age composition, and diagnostic definitions, particularly with regard to WC- and WHtR-based criteria. In addition, previous research has suggested that abdominal obesity among Chinese children and adolescents may have changed over time. For example, one study based on the China Health and Nutrition Survey reported increasing trends in abdominal obesity from 1993 to 2015 (5). However, the burden of abdominal obesity in Chinese youth has not been synthesized in a way that simultaneously quantifies overall prevalence, evaluates temporal patterns, and examines study-level correlates of heterogeneity.

Previous reviews and meta-analyses have mainly focused on BMI-defined overweight and general obesity among Chinese children and adolescents, rather than abdominal obesity (6, 7). Other studies have examined secular trends in abdominal obesity using specific national survey datasets, such as the China Health and Nutrition Survey, or have evaluated determinants of childhood obesity in China (5, 8, 9). Existing reviews of WHtR have mainly assessed diagnostic performance or optimal cut-off values for central obesity or cardiometabolic risk (10, 11). A dedicated synthesis of abdominal obesity prevalence among Chinese youth is therefore needed.

Therefore, the present study aimed to systematically review the available literature and conduct a meta-analysis to estimate the pooled prevalence of abdominal obesity among children and adolescents in China. Beyond providing an overall summary estimate, we sought to characterize study-level heterogeneity by sex, urban–rural setting, diagnostic criterion, and study quality, and to explore whether prevalence varied by survey year. By focusing specifically on WC- and WHtR-defined abdominal obesity, this review adds to previous evidence on BMI-defined obesity and provides information relevant to central adiposity surveillance in Chinese youth.

## 2 Methods

### 2.1 Study design and registration

This systematic review and meta-analysis was conducted in accordance with the Preferred Reporting Items for Systematic Reviews and Meta-Analyses (PRISMA) 2020 statement (12). The protocol for this review was prospectively registered in the International Prospective Register of Systematic Reviews (PROSPERO) under registration number CRD420251053503.

### 2.2 Search strategy

A comprehensive literature search was conducted in PubMed, Web of Science, Embase, CNKI, WanFang, and VIP from inception to December 31, 2025, to identify studies reporting the prevalence of abdominal obesity among children and adolescents in China. The search strategy combined controlled vocabulary and free-text terms related to abdominal obesity, children or adolescents, prevalence, and China. The detailed search strings for each database are provided in the Supplementary material.

The review methods were developed with reference to methodological guidance for systematic reviews of observational epidemiological studies reporting prevalence data (13).

### 2.3 Eligibility criteria

Studies were included if they met the following criteria:

1. The study population consisted of children and/or adolescents in China, defined in this review as participants aged 6–18 years.
2. The study was conducted in the general population, such as school-based, community-based, or population-based samples.
3. The study reported the prevalence of abdominal obesity, or provided sufficient data to calculate it.
4. Abdominal obesity was defined using WC, WHtR, or other clearly stated anthropometric criteria.
5. The study used an observational design, including cross-sectional surveys or baseline data from cohort studies.
6. The article was published in Chinese or English.

Studies were excluded if they:

1. Were reviews, comments, conference abstracts, case reports, or other non-original publications.
2. Did not report abdominal obesity outcomes or lacked sufficient data for pooling.
3. Involved special populations not representative of the general child or adolescent population. In this review, special populations were defined as participants selected on the basis of specific diseases, clinical conditions, treatment status, or other high-risk characteristics, such as children with diabetes, endocrine disorders, chronic kidney disease, genetic syndromes, severe disability, or hospital-based clinical samples.
4. Were conducted outside mainland China, Hong Kong, Macao, or Taiwan.
5. Used duplicate or overlapping datasets, in which case the most informative study or dataset was retained. The handling of overlapping reports is documented in Supplementary Table S1.
6. Included mixed age groups or mixed populations from which data for children and adolescents aged 6–18 years could not be extracted separately.

For studies with mixed samples, we applied the following rule. If a study included participants outside the age range of 6–18 years, it was included only when data for the eligible age group could be extracted separately or when the sample was clearly described as a school-age child/adolescent population and the age range only slightly exceeded the prespecified range. If adult, preschool, or special-population data were combined with child/adolescent data and could not be separated, the study was excluded.

### 2.4 Study selection and data extraction

All records identified through the literature search were imported into a reference management program, and duplicates were removed. Two reviewers independently screened titles and abstracts, followed by full-text review of potentially eligible articles. Disagreements were resolved through discussion and, when necessary, consultation with a third reviewer. When multiple reports used overlapping datasets, the study with the most complete information or the most appropriate analytic sample was retained.

Data were independently extracted by two reviewers using a standardized form. The following information was collected from each study: first author, publication year, survey year, study region, urban–rural setting, sample size, age range, sex, diagnostic criterion for abdominal obesity, number of abdominal obesity cases or prevalence estimate, and study quality score. The cutoff rules used in the included studies are summarized in Supplementary Table S2. Any discrepancies in data extraction were resolved by consensus.

### 2.5 Quality assessment

The methodological quality of the included studies was assessed independently by two reviewers using the Joanna Briggs Institute (JBI) Critical Appraisal Checklist for Studies Reporting Prevalence Data, which is commonly recommended for systematic reviews of prevalence studies (13). The checklist evaluates domains including sample frame, sampling method, sample size adequacy, description of participants and setting, data coverage, validity and reliability of outcome measurement, statistical analysis, and response rate.

For the present study, the total JBI score was used to categorize studies into three quality levels: high quality (8–9 points), moderate quality (6–7 points), and low quality (≤5 points).

### 2.6 Statistical analysis

All statistical analyses were conducted using Stata version 14.0 (StataCorp, College Station, TX, USA). Because this study synthesized prevalence estimates, meta-analysis of proportions was performed. Before pooling in Stata, the prevalence proportions were transformed using the Freeman–Tukey double arcsine transformation to stabilize variances. Random-effects meta-analysis was then performed on the transformed proportions, and the pooled prevalence estimates with corresponding 95% confidence intervals were back-transformed to the original proportion scale for presentation and interpretation. Pooled estimates were calculated using a random-effects model with the DerSimonian–Laird method to estimate between-study variance, because substantial methodological, clinical, and population-level heterogeneity was expected across studies. In addition to the 95% confidence interval of the pooled prevalence, a 95% prediction interval was calculated to reflect the expected range of prevalence estimates across comparable study settings while accounting for between-study heterogeneity.

Between-study heterogeneity was assessed using Cochran’s Q test and the I^2^ statistic. Prespecified subgroup analyses were conducted according to urban–rural setting, sex, diagnostic criterion, and study quality. These subgroup analyses compared pooled prevalence estimates across study-defined subgroups and were not intended to provide direct evidence of individual-level or population-level contrasts. Because very high heterogeneity was anticipated in prevalence studies, subgroup and meta-regression findings were interpreted primarily as exploratory analyses intended to characterize variation rather than to establish causal explanations.

To examine temporal patterns, univariable meta-regression was first performed with survey year as the independent variable after centering survey year at the mean survey year. Centering was used to improve the interpretability of the intercept, which then represented the estimated value at the mean survey year rather than at year zero. The slope estimate and its statistical inference were not affected by centering. Meta-regression was performed on Freeman–Tukey transformed prevalence estimates using the corresponding standard errors.

This analysis was exploratory and assumed a linear association between survey year and prevalence at the study level. Because prevalence estimates may change nonlinearly over time and may be influenced by study region, population composition, sampling design, and diagnostic criteria, the survey-year meta-regression was not intended to establish a definitive secular trend. In addition, an exploratory nonlinear analysis was conducted using spline terms for centered survey year with three knots placed at the 10th, 50th, and 90th percentiles of centered survey year.

A multivariable meta-regression model was then fitted to explore whether survey year, study quality, urban–rural setting, and diagnostic criterion were associated with abdominal obesity prevalence at the study level, while recognizing that residual confounding by unmeasured study-level factors could not be excluded. The number of studies with available data for each covariate was recorded, and the multivariable model was fitted using complete-case data. Mixed-setting studies were treated as a separate category where relevant. Because the number of studies was limited relative to the number of study-level predictors, the multivariable meta-regression was interpreted as exploratory.

Publication bias was assessed by visual inspection of the funnel plot and Egger’s test. A leave-one-out sensitivity analysis was conducted to examine the robustness of the pooled prevalence estimate. In addition, because subgroup analysis showed that pooled prevalence differed by study quality, we performed an additional sensitivity analysis excluding low-quality studies, defined as studies with JBI scores ≤5, to examine whether the overall pooled prevalence was influenced by lower-quality evidence.

## 3 Results

### 3.1 Literature search results

**Figure 1:**
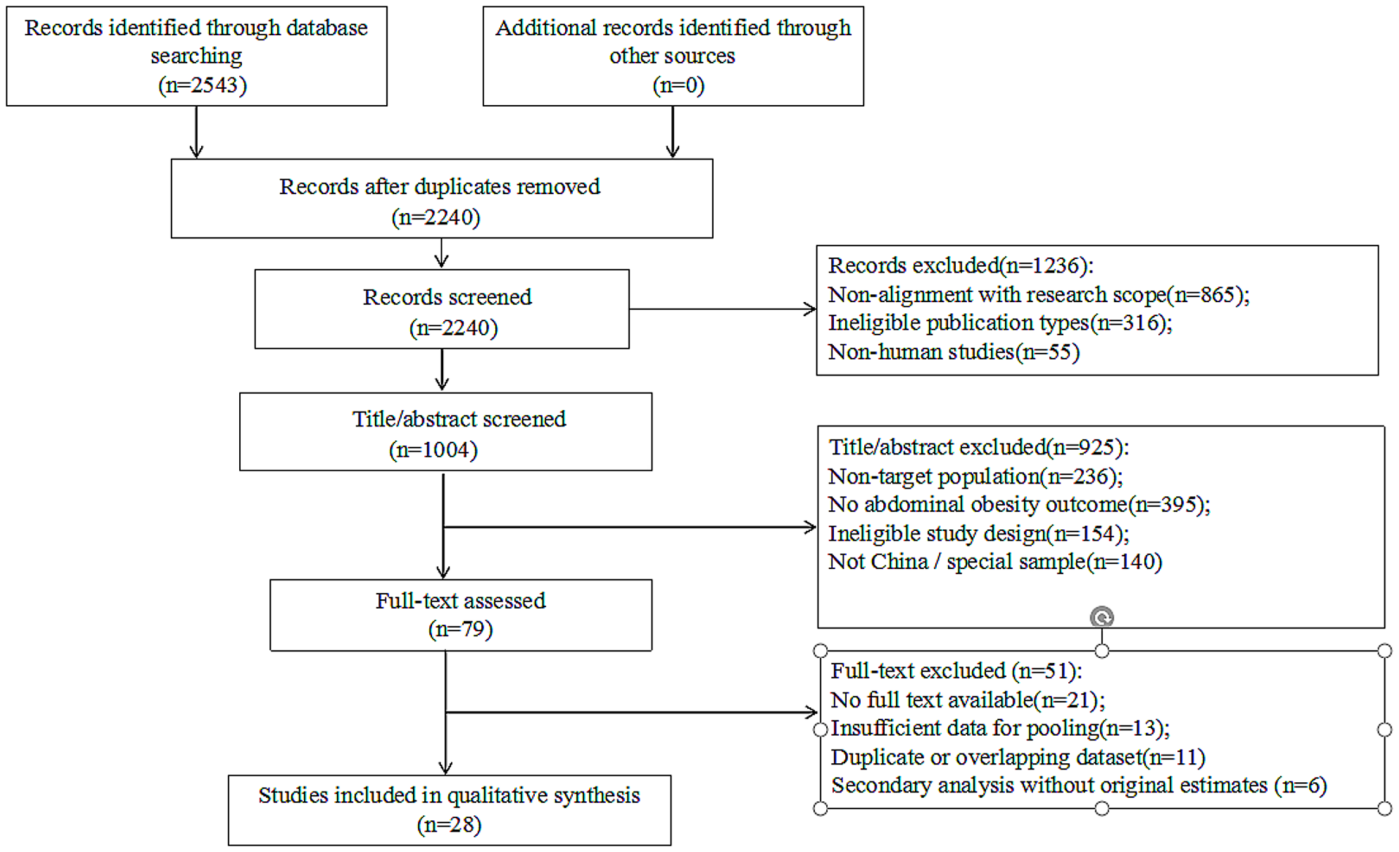
PRISMA flow diagram of study selection.

A total of 2,543 records were identified through database searching, and no additional records were identified from other sources. After removal of 303 duplicates, 2,240 records remained for screening. In the initial screening stage, 1,236 records were excluded because they were not aligned with the research scope, had ineligible publication types, or were non-human studies. The remaining 1,004 records underwent further title and abstract assessment, and 925 records were excluded due to non-target populations, absence of abdominal obesity outcomes, ineligible study designs, or studies conducted outside China/special samples. As a result, 79 full-text articles were assessed for eligibility. Of these, 51 articles were excluded for the following reasons: no full text available, insufficient data for pooling, duplicate or overlapping datasets, and secondary analyses without original estimates. Finally, 28 studies were included in the systematic review and meta-analysis (Figure 1) (14–41).

The included studies were published between 2015 and 2024, with survey years ranging from 2010 to 2023. The studies covered national surveys as well as regional investigations across multiple provinces and cities in China, including urban, rural, and mixed populations. Sample sizes ranged from 524 to 108,564 participants, and the age of participants generally ranged from 6 to 18 years. The reported prevalence of abdominal obesity varied substantially across studies, from 5.42 to 42.54%. Abdominal obesity was primarily defined using WC or WHtR, and the methodological quality of included studies ranged from 5 to 9 according to the JBI assessment (Supplementary Table S3).

### 3.2 Overall prevalence of abdominal obesity

**Figure 2:**
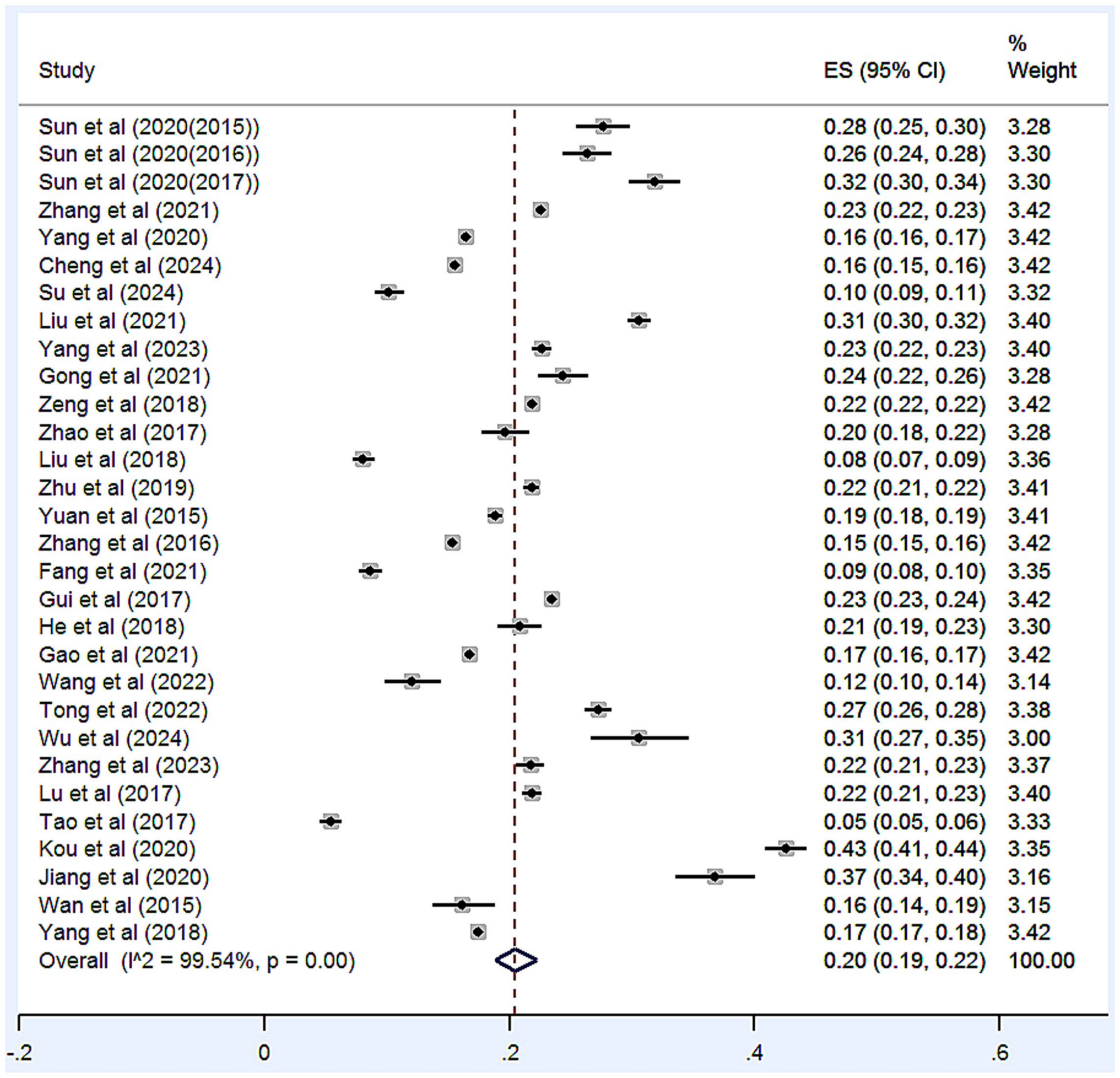
Forest plot of the pooled prevalence of abdominal obesity among children and adolescents in China.

The meta-analysis of 30 estimates from 28 studies showed that the pooled prevalence of abdominal obesity among children and adolescents in China was 20.0% (95% CI: 19.0–22.0%; 95% prediction interval: 11.9–31.3%). There was extremely high heterogeneity across studies (I^2^ = 99.54%, *p* < 0.001). The prediction interval indicates that the prevalence of abdominal obesity may vary substantially across different populations and study settings. Therefore, this pooled estimate should be interpreted as a summary across heterogeneous studies rather than as a precise national prevalence estimate. Detailed results are shown in Figure 2.

### 3.3 Subgroup analysis

Subgroup analyses were performed according to urban–rural setting, sex, diagnostic criterion, and study quality. These analyses compared pooled prevalence estimates across study-defined subgroups and should be interpreted as exploratory study-level analyses rather than conclusive evidence of between-subgroup differences.

The pooled estimate was 22.8% (95% CI: 20.6–25.1%) in studies or strata involving urban populations and 16.7% (95% CI: 13.3–20.4%) in those involving rural populations, with a *p* value of 0.006 for the subgroup test. By sex, the pooled estimate was 23.1% (95% CI: 20.9–25.4%) in male strata and 16.4% (95% CI: 14.5–18.5%) in female strata, with a p value <0.001 for the subgroup test. For diagnostic criteria, the pooled estimates were similar between studies using WHtR and those using WC (20.5% [95% CI: 18.1–23.0%] vs. 20.4% [95% CI: 17.9–23.1%]; P for subgroup test = 0.956). When stratified by study quality, the pooled estimates were 20.2% (95% CI: 17.4–23.2%) for studies with JBI scores of 8–9, 17.3% (95% CI: 15.1–19.6%) for those with scores of 6–7, and 27.4% (95% CI: 21.8–33.3%) for those with scores of ≤5, with a *p* value of 0.003 for the subgroup test. However, substantial heterogeneity remained within all subgroups, with I^2^ values ranging from 97.00 to 99.69% (Table 1). Therefore, these subgroup results should be viewed as descriptive patterns across study-level estimates rather than conclusive subgroup differences.

**Table 1:** Subgroup analyses of the pooled prevalence of abdominal obesity among children and adolescents in China.

Grouping method	Number of studies	Heterogeneity Test		Effect model	Meta-analysis (95% CI)	Inter-subgroup *P* value
		I^2^(%)	*P*			
Setting						
Urban	17	98.89	<0.001	Random	0.228 (0.206, 0.251)	0.006
Rural	6	99.07	<0.001	Random	0.167 (0.133, 0.204)	
Sex						
Male	20	99.29	<0.001	Random	0.231 (0.209, 0.254)	<0.001
Female	20	99.31	<0.001	Random	0.164 (0.145, 0.185)	
Diagnostic criteria						
WHtR	16	99.34	<0.001	Random	0.205 (0.181, 0.230)	0.956
WC	14	99.67	<0.001	Random	0.204 (0.179, 0.231)	
JBI						
8–9	11	99.69	<0.001	Random	0.202 (0.174, 0.232)	0.003
6–7	12	99.40	<0.001	Random	0.173 (0.151, 0.196)	
≤5	5	97.00	<0.001	Random	0.274 (0.218, 0.333)	

### 3.4 Exploratory meta-regression by centered survey year

**Figure 3:**
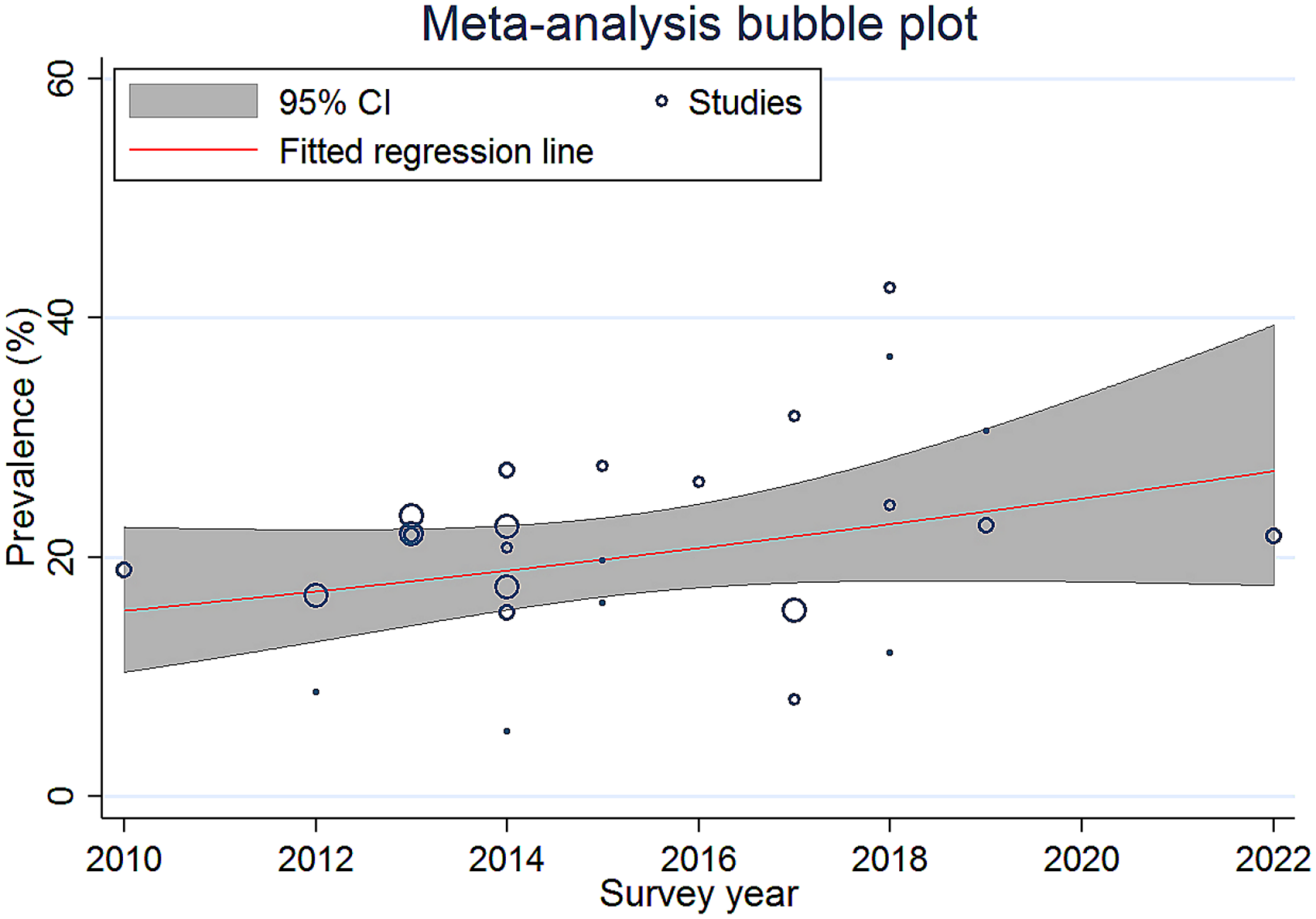
Exploratory meta-regression bubble plot of abdominal obesity prevalence by centered survey year.

An exploratory meta-regression analysis was performed to examine the study-level association between centered survey year and abdominal obesity prevalence. Survey year was centered at the mean survey year of 2015.33 to improve the interpretability of the intercept. In the univariable model, centered survey year was not significantly associated with abdominal obesity prevalence on the Freeman–Tukey transformed scale (coefficient = 0.0244, 95% CI: −0.0060 to 0.0548; *p* = 0.111). The slope estimate was unchanged compared with the model using raw survey year, whereas the intercept became interpretable as the estimated value at the mean survey year. The bubble plot showed considerable dispersion of prevalence estimates across survey years, without a clear linear pattern (Figure 3). Therefore, no reliable linear temporal pattern could be inferred from the available study-level data.

We further conducted an exploratory spline-based analysis using centered survey year with three knots. The knots corresponded approximately to survey years 2012, 2015, and 2019. The spline model was not statistically significant overall (F [2,24] = 1.32, *p* = 0.2859), and the nonlinear spline term was also not significant (*p* = 0.9246). Thus, this additional analysis did not provide evidence of a nonlinear temporal association. Because the included studies differed in region, population composition, sampling design, diagnostic criteria, and other study-level characteristics, the survey-year analysis should be interpreted cautiously. The results of the raw-year model, centered-year model, and exploratory spline model are summarized in Supplementary Table S4.

### 3.5 Multivariable meta-regression analysis

To further explore potential sources of heterogeneity beyond survey year, a primary multivariable meta-regression model was fitted. Data on survey year were available for 27 studies, whereas study quality, urban–rural setting, and diagnostic criterion were available for all 28 studies. Because the primary multivariable model used complete-case data, 27 studies contributed to each coefficient in the model. The model included four study-level predictors, corresponding to approximately 6.8 studies per predictor. Therefore, the model had limited statistical power and may have been overparameterized relative to the available number of studies.

As shown in Table 2, survey year, study quality, urban–rural setting, and diagnostic criterion were not significantly associated with the prevalence of abdominal obesity. The overall model was not statistically significant, and substantial residual heterogeneity remained. In an extended exploratory model additionally adjusting for male proportion and sample size, the results remained materially unchanged, but this model should be interpreted with particular caution because the number of predictors was larger relative to the available number of studies (Supplementary Table S5).

**Table 2:** Primary multivariable meta-regression analysis of study-level factors associated with abdominal obesity prevalence.

Covariate	Studies contributing to coefficient, k	β	SE	95% CI	*P*
Survey year	27	0.0459	0.0434	−0.0442, 0.1360	0.302
Study quality	27	0.0385	0.1040	−0.1773, 0.2543	0.715
Urban vs. mixed	27	0.3311	0.2494	−0.1861, 0.8482	0.198
WHtR vs. WC	27	−0.0520	0.2282	−0.5252, 0.4212	0.822

The primary multivariable meta-regression was fitted using complete-case data. Survey year was available for 27 studies, whereas study quality, urban–rural setting, and diagnostic criterion were available for all 28 studies; therefore, 27 studies contributed to each coefficient in the complete-case model. The model included four study-level predictors. tau^2^ = 0.308; I^2^res = 99.44%; adjusted R^2^ = −0.12%; model F = 0.99, *p* = 0.436.

In addition, important study-level factors such as geographic region, age distribution, socioeconomic context, sampling framework, and measurement protocol were not consistently available across studies and therefore could not be fully adjusted for in the model. Residual confounding is therefore likely.

### 3.6 Sensitivity analysis

**Figure 4:**
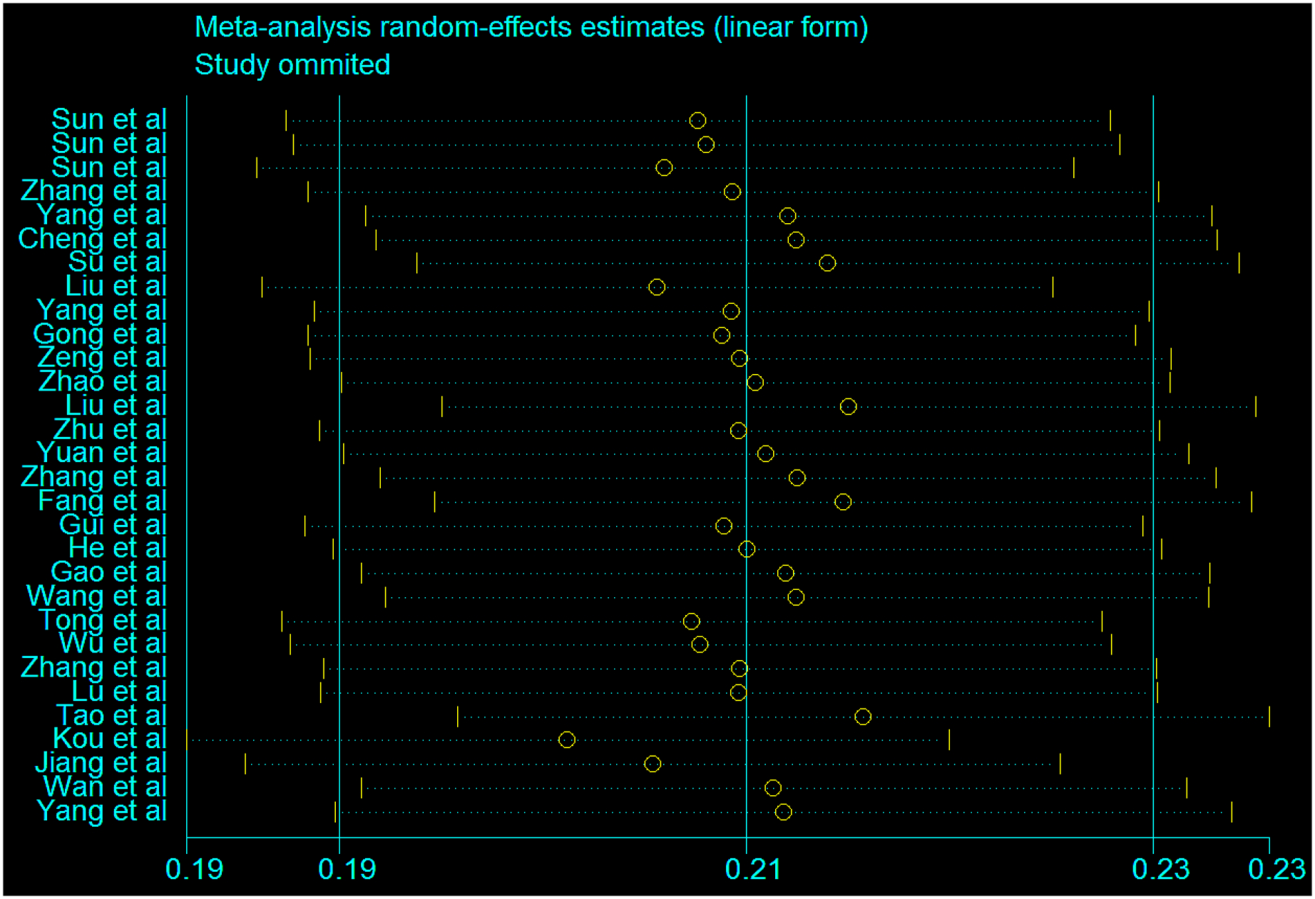
Leave-one-out sensitivity analysis of the pooled prevalence of abdominal obesity among children and adolescents in China.

A leave-one-out sensitivity analysis was conducted to assess the robustness of the pooled prevalence estimate of abdominal obesity. The results showed that omission of any single study did not substantially change the pooled estimate, and all recalculated prevalence estimates remained within a narrow range around the overall effect size (Figure 4). This indicates that no individual study exerted a decisive influence on the pooled prevalence, supporting the stability and robustness of the meta-analysis findings.

We further conducted a sensitivity analysis excluding low-quality studies with JBI scores ≤5. After exclusion of these studies, the pooled prevalence of abdominal obesity was 19.0% (95% CI: 18.0–21.0%), compared with 20.0% (95% CI: 19.0–22.0%) in the main analysis. This slight decrease indicates that the overall pooled estimate was not primarily driven by low-quality studies. However, because subgroup analysis showed a significant difference in pooled prevalence by study quality, methodological quality may have contributed to between-study variation.

### 3.7 Publication bias

**Figure 5:**
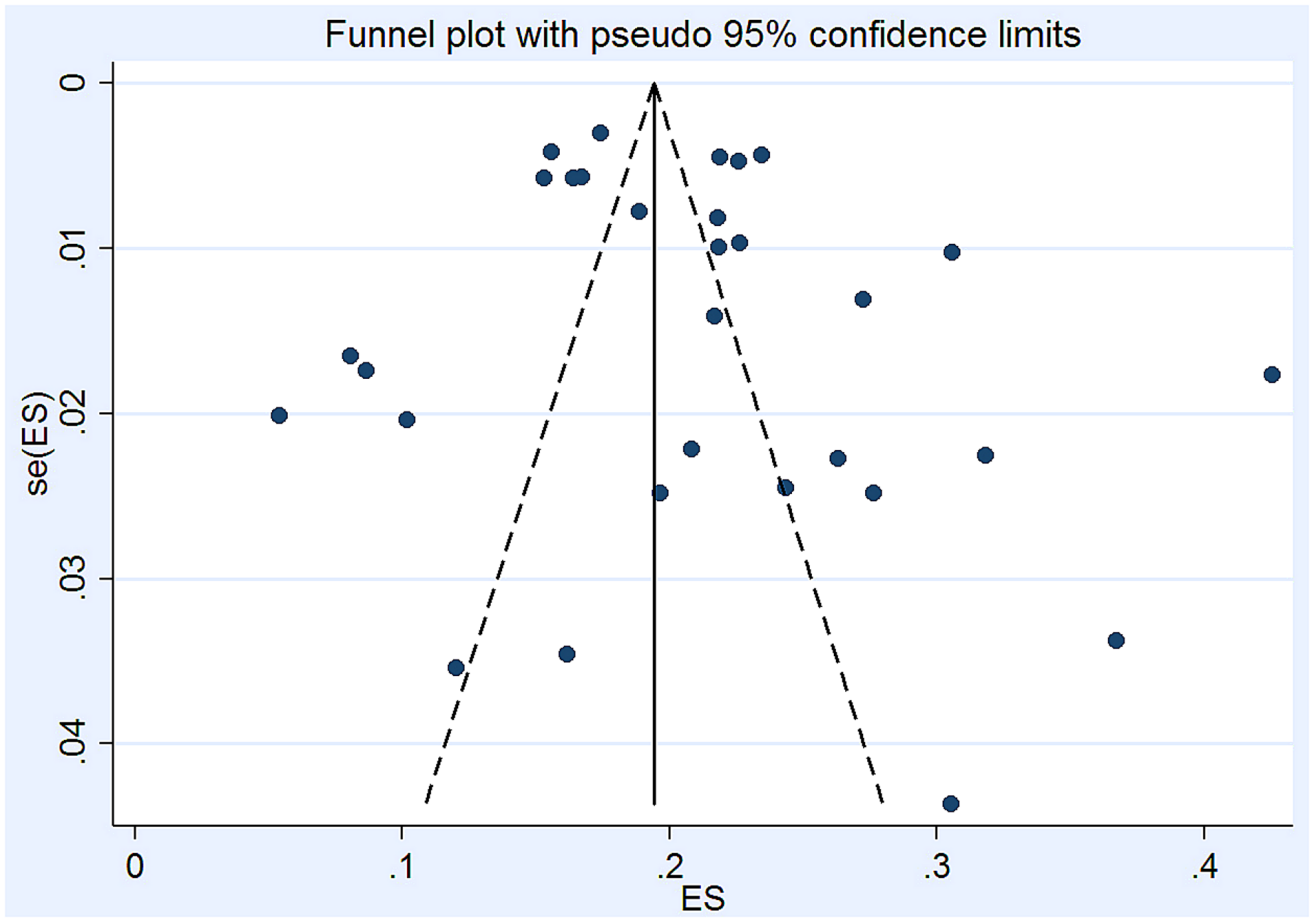
Funnel plot of publication bias for studies on abdominal obesity among children and adolescents in China.

Funnel plot asymmetry was not obvious on visual inspection, and Egger’s test did not provide statistical evidence of small-study effects (bias = 2.038, *p* = 0.253). However, these findings should be interpreted cautiously given the substantial between-study heterogeneity (Figure 5).

## 4 Discussion

In this systematic review and meta-analysis, we synthesized 30 prevalence estimates from 28 studies and found that the pooled prevalence of abdominal obesity among children and adolescents in China was 20.0% (95% CI: 19.0–22.0%; 95% prediction interval: 11.9–31.3%). The wide prediction interval and very high heterogeneity indicate substantial variation across study populations and methods. Therefore, the pooled estimate should be interpreted as a summary of heterogeneous study-level evidence rather than as a precise national prevalence estimate. Subgroup analyses showed higher pooled estimates in male and urban strata, while estimates were similar between WC- and WHtR-based definitions. However, because heterogeneity remained high within subgroups, these findings should be interpreted as exploratory study-level patterns rather than conclusive population-level contrasts.

Our findings should be interpreted within the broader context of nutrition transition in China. Over recent decades, rapid economic development and urbanization have altered dietary patterns, food environments, movement behaviors, and family lifestyles, contributing to increasing adiposity in children and adolescents (4). From a nutritional epidemiology perspective, abdominal obesity deserves particular attention because central adiposity is more closely linked to insulin resistance, dyslipidemia, and other cardiometabolic abnormalities than BMI alone, and thus may complement BMI-based surveillance in identifying young people at elevated metabolic risk (42). The pooled prevalence observed in our study suggests that the burden of central adiposity in Chinese youth is substantial enough to warrant routine consideration in prevention, nutrition monitoring, and school- and community-based health programs.

The higher pooled estimate in male strata is noteworthy, but it should be interpreted as a study-level pooled difference rather than direct evidence of a population-level sex contrast. This pattern is consistent with national obesity data from China and several original studies included in our review, which generally reported a higher burden of abdominal obesity among boys (4, 16, 18). Possible explanations include sex differences in body fat distribution, dietary behavior, screen use, sleep, physical activity, and pubertal development (4, 22, 43–45). However, these mechanisms were not directly tested in the present meta-analysis and should be regarded as hypotheses for future individual-level studies.

We also observed a higher pooled subgroup estimate in studies or strata involving urban populations than in those involving rural populations. This finding may reflect broader contextual differences related to urbanization, including changes in dietary patterns, food environments, physical activity, transport modes, and sedentary behavior (4, 46, 47). However, these mechanisms were not directly tested in our meta-analysis, and definitions of “urban” and “rural” varied across studies. Moreover, rural areas in China are also undergoing rapid nutrition and lifestyle transitions. The urban–rural finding should therefore be interpreted cautiously, particularly because multivariable meta-regression did not identify urban–rural setting as an independent source of heterogeneity.

The pooled estimates were similar between studies using WHtR and those using WC to define abdominal obesity. This result suggests that, at the population level, the overall prevalence estimates generated by the two approaches may be broadly comparable in Chinese children and adolescents. Prior evidence indicates that both WC and WHtR are useful markers of central adiposity and cardiometabolic risk in pediatric populations (2, 3). WHtR has often been considered particularly practical for screening because of its simplicity and age-related stability, whereas WC may require age- and sex-specific reference values. Our findings do not imply that the two indicators are interchangeable in all settings; rather, they suggest that differences in overall prevalence may be driven more by population composition and study context than by the broad choice between WC- and WHtR-based definitions.

With respect to temporal patterns, exploratory meta-regression using centered survey year did not identify a reliable linear association between survey year and abdominal obesity prevalence. The spline-based analysis also did not provide evidence of a nonlinear temporal association. These findings should not be interpreted as evidence that prevalence has not changed over time. Rather, differences in study region, population composition, sampling methods, age structure, diagnostic criteria, and measurement protocols may have obscured temporal patterns in the available study-level data.

High residual heterogeneity remained after subgroup analyses and multivariable meta-regression. This suggests that the variability in abdominal obesity prevalence was not adequately explained by the available study-level variables. The multivariable model included only 27 studies and four study-level predictors, resulting in limited statistical power. Geographic coverage, age distribution, pubertal stage, socioeconomic context, sampling framework, and measurement protocol were not consistently available and could not be fully adjusted for. For this reason, the subgroup and meta-regression analyses should be viewed as tools for describing variation rather than identifying causal determinants.

This study has several strengths. First, to our knowledge, this is one of the few systematic reviews and meta-analyses to comprehensively quantify the prevalence of abdominal obesity among children and adolescents in China while also examining temporal patterns and study-level correlates. Second, we incorporated subgroup analyses, sensitivity analysis, funnel plot assessment, Egger’s test, and both univariable and multivariable meta-regression, which provided a relatively comprehensive assessment of the available evidence. Third, our focus on abdominal obesity, rather than BMI-defined obesity alone, adds value for nutritional epidemiology and pediatric cardiometabolic risk surveillance.

Several limitations should also be acknowledged. First, the heterogeneity across studies was very high, which limits the interpretability of the pooled estimate and suggests substantial differences in underlying populations and methods. Second, subgroup analyses and meta-regression were based on study-level aggregate data rather than individual participant data; therefore, ecological bias cannot be excluded, and the results cannot support causal interpretation. Third, some included studies were not designed primarily as prevalence surveys, and a small number had lower methodological quality. Although excluding studies with JBI scores ≤5 yielded a similar pooled prevalence, methodological quality may still have contributed to between-study variation. Fourth, the main analysis used a random-effects model with the DerSimonian–Laird estimator; under extreme heterogeneity, uncertainty estimates may be sensitive to the choice of estimator. Therefore, our interpretation emphasized the prediction interval and descriptive patterns rather than precise inference. Fifth, despite the comprehensive search, the evidence base remained uneven across regions and years, and publication bias tests in meta-analyses of proportions should be interpreted cautiously.

The implications of our findings extend beyond obesity surveillance alone. Given that abdominal obesity affects about one in five Chinese children and adolescents, prevention efforts should begin early and should not rely solely on BMI-based screening. Routine assessment of central adiposity in schools, communities, and pediatric care may help identify high-risk children who might otherwise be missed by general obesity indicators alone. The higher burden observed in boys and urban populations suggests that interventions may need to be tailored to population subgroups and local environments. From a nutrition policy perspective, strategies that improve diet quality, reduce exposure to energy-dense and ultra-processed foods, discourage sugar-sweetened beverage consumption, and promote healthy daily routines may all contribute to reducing central adiposity in youth.

In conclusion, abdominal obesity is common among Chinese children and adolescents, with a pooled prevalence of approximately 20% across the included studies. Higher pooled estimates were observed in male and urban strata, but these subgroup findings should be interpreted cautiously because heterogeneity remained very high and the analyses were based on study-level data. More standardized surveillance methods, diagnostic criteria, and reporting practices are needed to improve comparability across studies and to better monitor central adiposity in Chinese youth.

## Data Availability

The original contributions presented in the study are included in the article/Supplementary material, further inquiries can be directed to the corresponding author.
