# Supplementary material for: Abdominal obesity among Chinese children and adolescents: a systematic review and meta-analysis of prevalence and study-level heterogeneity
Source: Front Nutr. 2026 Jul 29;13:1839333. doi: 10.3389/fnut.2026.1839333 (PMC13461509; doi:10.3389/fnut.2026.1839333)
Supplement: Supplementary file 1 [file table_1.docx]

**Supplementary Table S1. Handling of potentially overlapping datasets during full-text review**

| **No.** | **Data source / survey** | **Potentially overlapping report excluded after full-text review** | **Report retained in the meta-analysis** | **Basis for judging overlap** | **Reason for retaining this report** |
| --- | --- | --- | --- | --- | --- |
| 1 | China National Nutrition and Health Surveillance of Children and Lactating Mothers, 2016–2017 | Shi et al. Association of Dietary Patterns with Metabolic Syndrome in Chinese Children and Adolescents Aged 7–17 | Cheng et al., 2024 | Both reports used data from the China National Nutrition and Health Surveillance of Children and Lactating Mothers in 2016–2017 and included children/adolescents aged 7–17 years. | Cheng et al. was retained because it reported abdominal obesity as a study outcome and provided data more directly relevant to the present prevalence synthesis. |
| 2 | China National Nutrition and Health Surveillance of Children and Lactating Mothers, 2016–2017 | Shi et al. Nutrient Patterns and Its Association with Metabolic Syndrome among Chinese Children and Adolescents Aged 7–17 | Cheng et al., 2024 | Both reports were based on the same 2016–2017 national nutrition surveillance dataset and the same age range. | Cheng et al. was retained to avoid duplicate inclusion of the same surveillance population and because it provided clearer abdominal-obesity-related data for extraction. |
| 3 | China National Nutrition and Health Surveillance of Children and Lactating Mothers, 2016–2017 | Shi et al. Prevalence and Correlates of Metabolic Syndrome and Its Components in Chinese Children and Adolescents Aged 7–17 | Cheng et al., 2024 | Both reports used the same 2016–2017 national surveillance source and reported metabolic syndrome components, including abdominal obesity. | Cheng et al. was retained because it was the more recent report used for the main extraction and avoided repeated use of the same surveillance dataset. |
| 4 | China National Nutrition and Health Surveillance of Children and Lactating Mothers, 2016–2017 | Zhang et al. The Relationship between Birth Weight and the Risk of Overweight and Obesity in Children and Adolescents in China | Cheng et al., 2024 | Both reports used the same 2016–2017 national nutrition surveillance source. | Cheng et al. was retained because the excluded report focused mainly on birth weight and general obesity, whereas Cheng et al. provided abdominal-obesity-related data more suitable for the current review. |
| 5 | National school-based health lifestyles intervention baseline survey, China, 2013, NCT02343588 | Yang et al. Association of High Birth Weight with Overweight and Obesity in Chinese Schoolchildren | Yang et al., 2020 | Both reports used baseline data from the same national multicenter school-based project registered as NCT02343588. | Yang et al., 2020 was retained because it focused specifically on abdominal obesity and WHtR, whereas the excluded report mainly examined BMI-defined overweight/obesity. |
| 6 | National school-based health lifestyles intervention baseline survey, China, 2013, NCT02343588 | Wang et al. Relationship between Parental Overweight and Obesity and Childhood Metabolic Syndrome Risk in Their Offspring | Zhu et al., 2020 | Both reports used the same national multicenter school-based baseline dataset and examined metabolic syndrome-related outcomes in children and adolescents. | Zhu et al., 2020 was retained because it provided a broader description of metabolic syndrome and related components in Chinese children and adolescents, including abdominal obesity. |
| 7 | National school-based health lifestyles intervention baseline survey, China, 2013, NCT02343588 | Chen et al. A National School-Based Health Lifestyles Intervention among Chinese Children and Adolescents against Obesity: Rationale, Design and Methodology of a Randomized Controlled Trial in China | Zeng et al., 2018 | The design paper described the same national school-based intervention project from which later baseline analyses were derived. | Zeng et al., 2018 was retained because it reported extractable abdominal obesity prevalence estimates, whereas the excluded report was a protocol/design paper without usable prevalence data. |
| 8 | National school-based health lifestyles intervention baseline survey, China, 2013, NCT02343588 | Ma et al. Waist Circumference Is Better Than Other Anthropometric Indices for Predicting Cardiovascular Disease Risk Factors in Chinese Children | Zeng et al., 2018 | Both reports were linked to the same school-based obesity prevention research network and used overlapping anthropometric measurements in Chinese school-aged children. | Zeng et al., 2018 was retained because it included a larger national sample and provided abdominal obesity prevalence by WC and WHtR. |
| 9 | Childhood Obesity Study in China Mega-cities, 2015–2017 | Ma et al. A 3-Year Longitudinal Study of Pocket Money, Eating Behavior and Weight Status: The Childhood Obesity Study in China Mega-cities | Sun et al., 2021 | Both reports used data from the Childhood Obesity Study in China Mega-cities and included repeated measurements during 2015–2017. | Sun et al., 2021 was retained because it directly examined physical activity, sedentary behavior, and childhood obesity over 3 years and provided more suitable obesity-related outcome data for extraction. |
| 10 | Childhood Obesity Study in China Mega-cities, 2015–2017 | Ma et al. A 3-Year Longitudinal Study of Effects of Parental Feeding Practices on Child Weight Status: The Childhood Obesity Study in China Mega-cities | Sun et al., 2021 | Both reports used the same COCM longitudinal cohort and overlapping participants from the 2015–2017 survey waves. | Sun et al., 2021 was retained because it provided the more direct longitudinal analysis of physical activity, sedentary behaviors, and obesity outcomes. |
| 11 | Childhood Obesity Study in China Mega-cities, 2015–2017 | Xue et al. Longitudinal Effects of School Policies on Children's Eating Behaviors and Weight Status: Findings from the Childhood Obesity Study in China Mega-cities | Sun et al., 2021 | Both reports used longitudinal data from the same COCM study. | Sun et al., 2021 was retained because it was more directly aligned with the obesity outcome extraction required for this meta-analysis, whereas the excluded report focused mainly on school policies and eating behaviors. |

**Note:** When multiple reports were based on the same or substantially overlapping survey population, we retained the report that provided the most relevant and extractable abdominal obesity prevalence data for the present meta-analysis. Reports that were protocols, secondary analyses without extractable prevalence data, or analyses using the same participants with less directly relevant outcomes were excluded to avoid double counting.
