# Supplementary material for: Abdominal obesity among Chinese children and adolescents: a systematic review and meta-analysis of prevalence and study-level heterogeneity
Source: Front Nutr. 2026 Jul 29;13:1839333. doi: 10.3389/fnut.2026.1839333 (PMC13461509; doi:10.3389/fnut.2026.1839333)
Supplement: Supplementary file 2 [file table_2.docx]

PubMed检索策略

("Obesity, Abdominal"[Mesh] OR "abdominal obesity" OR "central obesity" OR "waist circumference" OR "waist-to-height ratio") AND ("Child"[Mesh] OR "Adolescent"[Mesh] OR child* OR adolescent* OR youth OR pediatric) AND ("Prevalence"[Mesh] OR prevalence OR incidence) AND ("Risk Factors"[Mesh] OR risk factors OR determinants OR associations) AND ("China"[Mesh] OR China OR Chinese) Filters: ****from 2010 - 2025****

知网检索策略：

****(SU=(腹型肥胖+向心性肥胖+中心性肥胖) OR AB=(腰围+超标+异常+腰高比+腰腎比)) AND (SU=(儿童+青少年+学龄儿童) OR AB=(未成年人+少年)) AND(SU=(患病率+危险因素+横断面研究))AND(SU=(患病率+危险因素+横断面研究))****

Filters: ****from 2010 - 2025****
